# Kisspeptins and RFRP-3 Act in Concert to Synchronize Rodent Reproduction with Seasons

**DOI:** 10.3389/fnins.2013.00022

**Published:** 2013-02-26

**Authors:** Valérie Simonneaux, Caroline Ancel, Vincent Joseph Poirel, François Gauer

**Affiliations:** ^1^Institut des Neurosciences Cellulaires et Intégratives, CNRS UPR 3212Strasbourg, France

**Keywords:** seasonal reproduction, melatonin, kisspeptin, RFRP-3, syrian hamster

## Abstract

Seasonal mammals use the photoperiodic variation in the nocturnal production of the pineal hormone melatonin to synchronize their reproductive activity with seasons. In rodents, the (SD) short day profile of melatonin secretion has long been proven to inhibit reproductive activity. Lately, we demonstrated that melatonin regulates the expression of the hypothalamic peptides kisspeptins (Kp) and RFamide-related peptide-3 (RFRP-3), recently discovered as potent regulators of gonadotropin-releasing hormone (GnRH) neuron activity. In the male Syrian hamster, Kp expression in the arcuate nucleus is down-regulated by melatonin independently of the inhibitory feedback of testosterone. A central or peripheral administration of Kp induces an increase in pituitary gonadotropins and gonadal hormone secretion, but most importantly a chronic infusion of the peptide reactivates the photo-inhibited reproductive axis of Syrian hamsters kept in SD conditions. RFRP-3 expression in the dorsomedial hypothalamus is also strongly inhibited by melatonin in a SD photoperiod. Although RFRP-3 is usually considered as an inhibitory component of the gonadotropic axis, a central acute administration of RFRP-3 in the male Syrian hamster induces a marked increase in gonadotropin secretion and testosterone production. Furthermore, a chronic central infusion of RFRP-3 in SD-adapted hamsters reactivates the reproductive axis, in the same manner as Kp. Both Kp and RFRP-3 neurons project onto GnRH neurons and both neuropeptides regulate GnRH neuron activity. In addition, central RFRP-3 infusion was associated with a significant increase in arcuate Kp expression. However, the actual sites of action of both peptides in the Syrian hamster brain are still unknown. Altogether our findings indicate that Kp and RFRP neurons are pivotal relays for the seasonal regulation of reproduction, and also suggest that RFRP neurons might be the primary target of the melatoninergic message.

## Melatonin Drives Seasonal Reproduction

Living on earth imposes daily and annual changes in light, temperature, and humidity, which in turn cause cyclic changes in food availability. Organisms therefore must anticipate these predictable changes and adapt their biological functions to ensure their survival. Specifically, most mammals restrict their fertility to a particular time of the year to ensure that the birth and weaning of the offspring occur during the most favorable season. Although various environmental factors are important to consider, a majority of mammalian species use the highly predictable annual variations in light duration (or photoperiod) to establish the time of year and adapt their reproductive activity accordingly.

The photoperiod is converted into neuroendocrine signals via a dedicated photoneuroendocrine pathway, which involves the master biological clock located in the suprachiasmatic nuclei and other hypothalamic nuclei, which in turn synchronize various biological activities with the time of the day and year (Buijs and Kalsbeek, 2001; Kalsbeek et al., 2006). One neuronal pathway controls the pineal gland which releases the hormone melatonin exclusively at night, with a duration depending on the length of the night. Therefore, photoperiodic variations in circulating levels of melatonin throughout the year provide the body with a robust and reproducible representation of the seasons (Simonneaux and Ribelayga, 2003). Since the pioneer studies of Reiter and Hoffman in the 1960s, it is well established that the annual/photoperiodic rhythm in nocturnal melatonin is critical to synchronize reproduction with the seasons (Hoffman and Reiter, 1965; Bartness et al., 1993; Pitrosky and Pevet, 1997). Thus, small rodents like the Syrian or Siberian hamster are sexually active when kept under long day (LD) conditions. Upon exposure to short day (SD) conditions for 8–10 weeks, they undergo a dramatic inhibition of their reproductive activity manifested by a decrease in levels of circulating gonadotropins and a marked involution of the gonads and accessory organs, resulting in low levels of circulating sex steroids. Surgical removal of the Syrian hamster’s pineal gland before exposure to SD prevents their sexual inactivation. Conversely, exogenous melatonin injections mimicking SD conditions in hamsters maintained in LD induce sexual inactivation. Notably, the photoperiodic change in circulating melatonin may induce different effects according to the animal’s reproductive physiology. Thus, sheep are sexually active in SD and become quiescent after transfer to LD and yet the melatonin signal synchronizes reproductive activity according to day length. Hamsters are termed LD breeders whereas sheep are SD breeders, and due to different durations of gestation, the offspring of both kinds of breeders is born in spring/early summer, when environmental conditions are most favorable for offspring survival. So far, it is still unknown how the reproductive system of LD and SD breeders responds oppositely to the same melatoninergic message.

Actually, the cellular sites of melatonin action for the seasonal control of reproductive activity are still unknown. Melatonin binding sites have been identified in a number of brain structures but with considerable species-differences (Masson-Pevet et al., 1994). Lesion or melatonin-infusion studies at various anatomical sites suggested that the mediobasal hypothalamus is involved in melatonin’s effects on reproduction (Malpaux et al., 1993; Maywood and Hastings, 1995). Besides, a high density of melatonin receptors has been identified in the pars tuberalis of the adenohypophysis in a number of mammalian species (Masson-Pevet and Gauer, 1994), and recent studies point to this structure as a critical site for the effect of melatonin on seasonal functions, notably reproduction (Hanon et al., 2008; Nakao et al., 2008; Dardente et al., 2010).

A few years ago, taking the opportunity of the discovery of new neuropeptides involved in the central regulation of reproductive activity, namely kisspeptins (Kp) and RFRPs, we chose to develop a backwards approach to delineate how melatonin may synchronize reproduction in seasonal rodents.

## Kisspeptins are a Pivotal Activators of GnRH Neurons

In mammals, early studies on the regulation of the reproductive axis have emphasized the pivotal role of the pulsatile release of gonadotropin-releasing hormone (GnRH), a decapeptide synthesized by a small population of neurons located in the rostral hypothalamus, mainly the preoptic area (POA), and released principally into the pericapillary space of the pituitary portal system at the median eminence (Hahn and Coen, 2006). GnRH drives the secretion of gonadotropins, luteinizing (LH), and folliculo-stimulating (FSH) hormones, by the pituitary, which in turn control gametogenesis and the production of sex steroids by the gonads. Finally, sex steroids feedback on the gonadotropic axis to maintain the homeostasis of the reproductive system under different physiological conditions (Witkin et al., 1982). Recently, a major advance in our understanding of the neuronal mechanisms controlling GnRH secretion came with the identification of new hypothalamic peptides, namely Kp.

In 2003, the discovery that loss-of-function mutations of the gene encoding the Kp receptor *Kiss1r/GPR54* in humans and rodents (de Roux et al., 2003; Seminara et al., 2003) prevent pubertal development and cause infertility spurred great interest in the role and mechanism of action of Kps in reproductive function (Pinilla et al., 2012 for review). In mammals, the *Kiss1* gene is translated into a 145-amino acid (aa) precursor further processed into Kp fragments of smaller sizes (from 54 aa in humans or 52 aa in rodents down to 10 aa). All fragments share the same 10 amino acid C-terminal sequence with a final amidated Arg-Phe-(or Tyr in rat and mice)-NH_2_ motif, a hallmark of the RFamide super family of peptides (Kotani et al., 2001). Shortly after the seminal finding that *Kiss1r* mutations lead to reproductive defects, several studies reported that low doses of central Kp10 or Kp54 display a very potent stimulatory effect on LH and FSH secretion in a variety of mammalian species, including rodents, sheep, primates, and humans (Gottsch et al., 2004; Dhillo et al., 2005; Navarro et al., 2005a,b; Shahab et al., 2005; Caraty et al., 2007; Mikkelsen et al., 2009). Actually, the common 10 C-terminal amino acid sequence appears sufficient to induce a full activation of the Kiss1r. However, it is not yet known what is (are) the naturally occurring endogenous form(s) of the peptide.

The *Kiss1* gene is expressed mainly in two hypothalamic areas in rodents, the arcuate nucleus (ARC), and a more rostral area, the anteroventral periventricular nucleus (AVPV; Gottsch et al., 2004; Smith et al., 2005a,b; Kauffman et al., 2007; Clarkson et al., 2009; Mikkelsen and Simonneaux, 2009; Ansel et al., 2010), and in the POA in sheep (Franceschini et al., 2006), but also in rodent amygdala (Cravo et al., 2011). In rodents, the AVPV *Kiss1* neuronal population displays a marked sexual dimorphism, being larger in females than in males. This is in line with the pivotal role of AVPV Kp in the control of the preovulatory LH surge (Kinoshita et al., 2005; Pineda et al., 2010a). Kp neurons project to the POA where GnRH neurons are located (Clarkson and Herbison, 2006; Clarkson et al., 2009; Desroziers et al., 2010). Most GnRH neurons (>90%) express Kiss1R and administration of Kp activates 90% of GnRH neurons and induces GnRH release (Irwig et al., 2004; Han et al., 2005; Messager et al., 2005). However, there is some evidence that Kp may activate GnRH neurons via intermediary cells (Hanchate et al., 2012). Additionally, Kp fibers extend toward the median eminence where the peptide acts on GnRH nerve terminals to regulate GnRH release (d’Anglemont de Tassigny et al., 2008). As a consequence, peripheral Kp administration (via intravenous, intraperitoneal, subcutaneous routes) is also efficient in stimulating gonadotropin secretion, and this further emphasizes the potential application of Kp in the medical and agronomical fields. Finally Kp-immunoreactive fibers, especially arising from the ARC, project to other hypothalamic and limbic areas indicating that the peptide may (might? Pour que ce soit plus hypothétique?) be involved in non-reproductive functions (Kinoshita et al., 2005; Clarkson and Herbison, 2006; Clarkson et al., 2009; Mikkelsen and Simonneaux, 2009; Desroziers et al., 2010). Unfortunately, the understanding of Kp central sites of action is weak since a highly selective Kiss1r antibody is still lacking. A few studies addressing the sites of *Kiss1R* mRNA expression by *in situ* hybridization, PCR amplification, or more recently using a *Kiss1r* LacZ knock-in mouse model (Lee et al., 1999; Irwig et al., 2004; Navarro et al., 2004; Herbison et al., 2010) confirm the localization of Kiss1R in GnRH neurons, but also in other discrete hypothalamic and extra-hypothalamic areas.

Strikingly, Kp neurons are the main target for the positive (in AVPV/POA) and negative (in ARC) central feedback effects of sex steroids (Smith et al., 2005a,b; Revel et al., 2006; Ansel et al., 2010), underlying their pivotal role in the tightly controlled positive/negative feedback loops of the reproductive axis. In addition, studies have also reported that metabolic factors, particularly leptin, impact on Kp expression to participate in the regulation of reproductive function (Smith et al., 2006a; Castellano et al., 2010).

## Kisspeptins Mediate the Photoperiodic Control of Reproduction in Seasonal Breeders

As stated above, reproductive function in many mammalian species is highly sensitive to environmental cues, to adapt the time of birth to seasonal changes in temperature and food availability. Given the pivotal role of Kp neurons in the control of reproductive activity, we and other groups investigated whether and how Kp neurons may gate seasonal reproduction.

Using the male Syrian hamster as a model for the study of seasonal reproduction, we tested the hypothesis that photoperiod/melatonin could regulate this function via an action on Kp neurons (Revel et al., 2006, 2007; Ansel et al., 2010, 2011). We reported that *Kiss1* mRNA and Kp-ir in the ARC and AVPV are significantly down-regulated when male Syrian hamsters are kept for 8–10 weeks in SD, in parallel to the expected important reduction in testis size and circulating levels of testosterone, as compared to LD conditions (Revel et al., 2006). In the ARC, the SD-induced reduction in *Kiss1* expression was not the result of decreased levels of testosterone since castration of LD Syrian hamsters led to a significant increase in *Kiss1* mRNA levels in the ARC (Revel et al., 2006; Ansel et al., 2010). This observation is in agreement with the reported inhibitory effect of sex hormones on *Kiss1* expression in the ARC. Importantly, we observed that the removal of endogenous melatonin by pinealectomy in SD-exposed Syrian hamsters increased ARC *Kiss1* mRNA and reactivated reproductive activity within a few weeks. Conversely, late afternoon melatonin injections in sexually active Syrian hamsters kept in LD significantly reduced ARC *Kiss1* mRNA levels, together with a marked reduction in testicular activity (Revel et al., 2006; Ansel et al., 2010). It is important to note that melatonin treatment had to be carried out for at least 5 weeks to be effective. In the AVPV, the level of *Kiss1* mRNA was also reduced in SD compared to LD. However, because testosterone stimulates AVPV *Kiss1* gene expression, the SD-induced decrease in *Kiss1* mRNA is a consequence of melatonin/SD-induced reduction in the circulating level of testosterone (Ansel et al., 2010). These findings strongly suggest that the long nocturnal peak of melatonin in SD inactivates reproductive activity via a sex steroid-independent (ARC) or a sex steroid-related (AVPV) inhibition of *Kiss1* expression in the male Syrian hamster. Strikingly, when Syrian hamsters became refractory to the inhibitory melatoninergic signal (kept over 25 weeks in SD), the spontaneous sexual reactivation was associated with an increase in *Kiss1* mRNA in the ARC, indicating that *Kiss1* expression also escapes melatonin’s inhibitory signal in photorefractory animals (Revel et al., 2006, 2007).

In order to test whether the photoperiodic variation in hypothalamic Kp is critical for the seasonal control of reproductive activity, sexually inactive SD Syrian hamsters received a chronic intracerebroventricular infusion of 0.25 nmol/h Kp10 or vehicle for 4 weeks. Remarkably, the Kp-treated animals underwent a full reactivation of their testicular activity, and levels of circulating testosterone increased up to values observed in SD hamsters transferred back to LD on the day of surgery (Revel et al., 2006). Furthermore, a peripheral administration of Kp54 (acute intraperitoneal twice a day injections, but not a continuous subcutaneous administration) in SD photo-inhibited hamsters could restore testicular activity as well (Ansel et al., 2011). Altogether, our data demonstrate that in Syrian hamsters, melatonin in SD inhibits Kp expression in the ARC and AVPV which in turn down-regulates reproductive activity.

The stimulatory effect observed when Kp are given centrally or systemically indicates that Kp act on GnRH cell bodies and nerve terminals to activate Syrian hamster reproductive activity. At this point however, the actual cellular targets for Kp in the Syrian hamster brain are not identified and are difficult to determine in the absence of a Kp radioligand or selective Kiss1r antibody.

In an attempt to disclose the putative sites of action of Kp, we first cloned the Syrian hamster *Kiss1r* and mapped the location of *Kiss1r* gene expression by *in situ* hybridization in the brain of male hamsters kept in either LD or SD conditions.

For *Kiss1r* cloning, total RNA was extracted from Syrian hamster hypothalamus using RNAble (Eurobio) according to the protocol of Chomczynski and Sacchi (1987) and reverse transcribed. Hamster *Kiss1r* amplicons were obtained using primers designed to frame a specific region of the *Kiss1R* cDNA based on the previously published rat sequence (AF115516, 328–638 bp; 5′ CTGGGCGACTTCATGTGCAAGTT 3′ and 5′ TATAGGGCCAGCAGGTTGTAGAG 3′). PCR amplified products of the predicted size (263-bp) were purified and ligated into the pCR2.1 cloning vector (Invitrogen). Identity of the clone was confirmed by sequencing (AGOWA) and the obtained sequence was deposited in GenBank (accession number GQ872419). The Syrian hamster *Kiss1r* cloned sequence shares 94 and 92% identity with the corresponding sequence of the known *Kiss1r* genes of mice and rats, respectively.

*In situ* hybridization with both sense and antisense [^35^S]UTP-labeled *Kiss1r* riboprobes was performed on 20 μm thick frozen hamster brain sections containing the main part of the diencephalon. Tissue sections were submitted to prehybridization treatments before an overnight hybridization of the riboprobe at 54°C in a 2X SSC/50% formamide hybridization medium. The posthybridization consisted in an RNAse (Sigma) treatment and a final high stringency wash in 0.01X SSC at 56°C to eliminate most of the non-specific labeling. The hybridized sections were subsequently exposed to X-ray films for 10 days and scanned for quantitative analysis.

**Figure 1:**
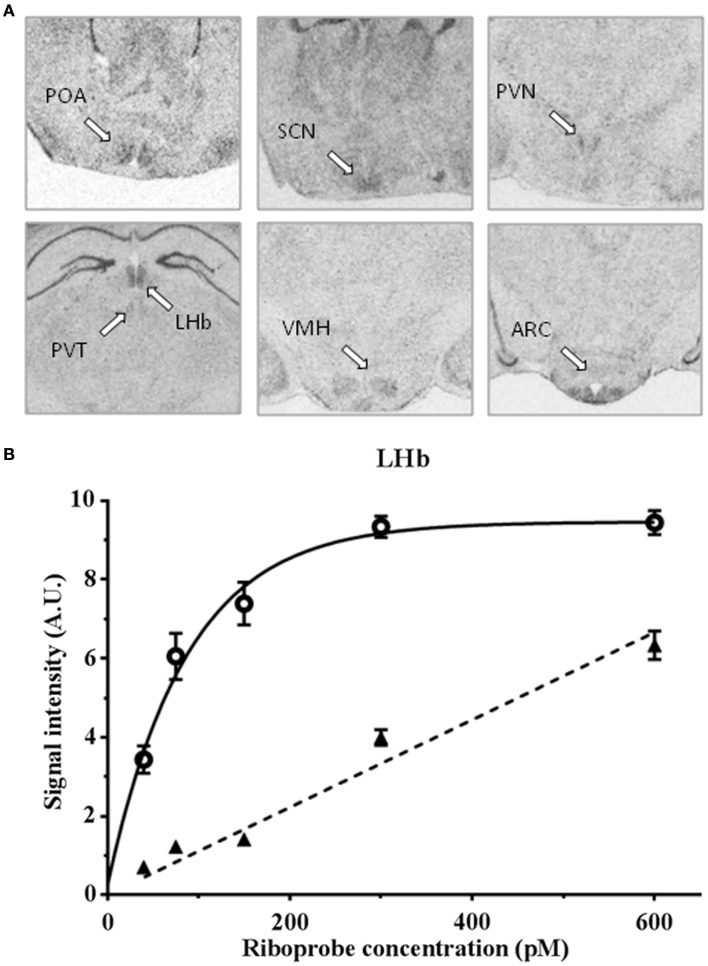
**(A)** *In situ* hybridization of a ^35^S-labeled antisense *Kiss1r* cRNA riboprobe in the preoptic area (POA), suprachiasmatic nucleus (SCN), paraventricular nucleus (PVN), lateral habenular nuclei (LHb), paraventricular thalamic nuclei (PVT), ventromedial hypothalamic nucleus (VMH), and arcuate nucleus (ARC) of Syrian hamster brain sections, bar = 2 mm; **(B)** specific (full line) and non-specific (dotted line) hybridization signal with a ^35^S-labeled antisense *Kiss1r* cRNA riboprobe in Syrian hamster lateral habenular nuclei.

**Figure 2:**
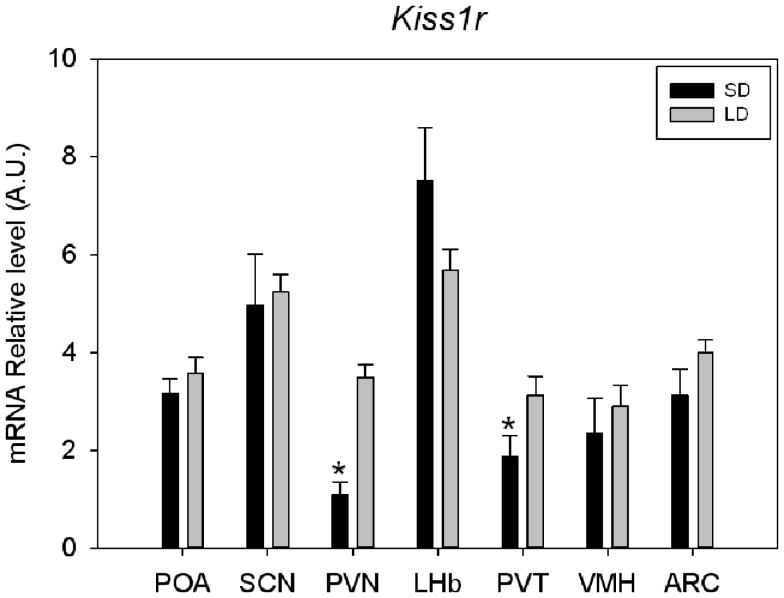
**Quantification of *Kiss1r* mRNA expression in various brain areas of Syrian hamsters housed in long (LD: 14 h light-10 h dark) or short (SD: 10 h light-14 h dark) day**. For each structure SD value was compared to LD value and considered as significant (*) for *p* < 0.05. suprachiasmatic nucleus (SCN), paraventricular nucleus (PVN), lateral habenular nucleus (LHb) parventricular nucleus of the thalamus (PVT), ventromedial hypothalamic nucleus (VMH), and arcuate nucleus (ARC).

A clear labeling with the antisense riboprobe was observed in various hypothalamic areas, particularly in the POA, the suprachiasmatic nucleus, the paraventricular nucleus, the ventromedial hypothalamic nucleus, the ARC, but also in the lateral habenular nucleus and paraventricular nucleus of the thalamus (Figure 1). No signal could be detected with the sense probe in the same cerebral structures. In contrast, hippocampus was labeled both with the antisense and sense probes. The specificity of the antisense hybridization probe was further demonstrated by experiments showing that the hybridization between the riboprobe and the targeted RNAs is saturable (Figure 1). Some of these structures were previously reported to express *Kiss1r* mRNA, the ARC in rats, and rhesus monkeys (Lee et al., 1999; Shahab et al., 2005), the ventromedial hypothalamic nucleus in monkeys (Shahab et al., 2005), and the lateral habenular nucleus in mice and rats (Lee et al., 1999; Herbison et al., 2010). In the POA, *Kiss1r* mRNA has been previously demonstrated to be located in GnRH neuron-containing areas of numerous species (Pinilla et al., 2012 for review). On the other hand, *Kiss1r* mRNA expression has not been reported so far in the hypothalamic suprachiasmatic and paraventricular nuclei and the thalamic paraventricular nucleus of other mammals. Interestingly, in both the hypothalamic and thalamic paraventricular nuclei, *Kiss1r* mRNA levels are significantly higher in LD as compared to SD in Syrian hamsters (Figure 2), and it will be interesting to test whether this is caused by higher levels of circulating testosterone in LD conditions. In order to understand the role of Kiss1r in these various structures, further studies are now required, particularly the phenotyping of the *Kiss1r*-expressing cells in the Syrian hamster brain.

Other seasonal species were also examined to investigate the regulation and role of Kp in reproduction. Thus, photoperiodic variation of Kp expression was reported in the Siberian hamster (Greives et al., 2007, 2008; Mason et al., 2007; Simonneaux et al., 2009). In sexually active male Siberian hamsters kept in LD, the number of Kp-ir neurons is larger in the AVPV but, unexpectedly, lower in the ARC as compared to SD-adapted animals. In this species, the photoperiodic regulation of Kp expression is strongly regulated by the feedback effect of sex steroids. Hence in LD sexually active animals, the high levels circulating testosterone inhibit Kp expression in the ARC and increase Kp expression in the AVPV. Nevertheless, in Siberian hamsters with a constant level of sex steroids (following castration or testosterone implant) photoperiod still impacts on Kp expression. Our hypothesis is that this photoperiodic, sex steroid-independent regulation may be more critical at the early phases of photoperiodic changes. Acute Kp10 injection in the male Siberian hamster triggers LH release but so far attempts at chronic peripheral administration of Kp10 could not reactivate the SD-inhibited reproductive axis.

In contrast to hamsters, sheep are reproductively active in SD and become quiescent as days become longer. But, similar to hamsters, this seasonal synchronization of reproduction is driven by melatonin. The photoperiodic regulation and effect of Kp have also been examined in the sheep (Franceschini et al., 2006; Caraty et al., 2007; Smith et al., 2008b; Chalivoix et al., 2010). The number of Kp neurons in the ARC and Kp-ir contacts onto GnRH neurons is higher in the SD breeding season as compared to LD non-breeding season, even in ovariectomized ewes (Franceschini et al., 2006; Caraty et al., 2007; Smith et al., 2008b; Chalivoix et al., 2010). This suggests that, similar to hamsters, both a steroid-independent and -dependent seasonal modulation of *Kiss1* expression occurs in the ovine ARC to drive seasonal reproduction. However, whether photoperiod impacts (in)directly on the kisspeptin system via melatonin has not been demonstrated so far. Importantly, Kp infusion during the anestrous season caused ovulation in more than 80% of treated animals whereas <20% of control animals ovulated, indicating that in sheep like in hamsters, Kp are a critical component of the seasonal regulation of reproductive activity (Caraty et al., 2007).

Collectively, the data acquired in seasonal breeders demonstrate that Kp expression is regulated by photoperiod. Because of the potent stimulatory effect of Kp on GnRH, this control appears critical to synchronize reproductive activity with the seasons. However, photoperiodic regulation of Kp expression is complex and appears to be species-dependent. In the Syrian hamster, the SD melatonin signal inhibits Kp expression, but this effect is modulated by the stimulatory (in the AVPV) and inhibitory (in the ARC) feedback effect of sex steroids. In the Siberian hamster, sex steroid feedback appears more potent than that of melatonin to control Kp expression. In the sheep, as opposed to hamsters, Kp expression is inhibited in LD when animals are sexually quiescent. To complicate matters some more, metabolic factors like leptin or food resources, also influenced by the seasons, impact on Kp expression (Smith et al., 2006b; Castellano et al., 2010; Matsuzaki et al., 2011; Quennell et al., 2011). Further investigations should determine how seasonal alterations of metabolic factors, melatonin, and sex steroids impact on reproductive activity via the Kp neurons.

In conclusion, the photoperiodic variation in circulating melatonin is part of the mechanism regulating hypothalamic Kp expression, to finely tune reproductive activity with the organism’s physiological conditions. However, it is yet unknown where melatonin is acting to control Kp expression. As Kp neurons, or areas where Kp neurons are located, do not contain melatonin receptors (Masson-Pevet et al., 1994; Li et al., 2011), melatonin is likely acting upstream of Kp neurons.

## RFRP-3 is Another RFamide Peptide Regulating the Gonadotropic Axis

Other peptides of the RFamide family characterized by a common C-terminal LPXRFamide (X = L or Q) motif were shown to regulate reproductive activity in birds and mammals (Hinuma et al., 2000; Tsutsui et al., 2000; Ukena and Tsutsui, 2001; Kriegsfeld et al., 2006). In the quail, one such peptide acts directly at the pituitary to inhibit gonadotropin release and is accordingly named gonadotropin-inhibitory hormone (GnIH; Tsutsui et al., 2000). In mammals, the *RFamide-related peptide* (*rfrp*) gene is expressed in neurons located in the mediobasal hypothalamus and it encodes a precursor that produces two peptides, RFRP-1 and RFRP-3 (Kriegsfeld et al., 2006; Clarke et al., 2008; Dardente et al., 2008; Revel et al., 2008; Smith et al., 2008a; Rizwan et al., 2009). RFRP neurons project to multiple brain regions including the POA, the ARC, the lateral septum, the anterior hypothalamus, and the bed nucleus of the stria terminalis (Ukena and Tsutsui, 2001; Kriegsfeld et al., 2006; Johnson et al., 2007; Mason et al., 2010). Notably, RFRP-immunoreactive fibers make apparent contacts with 20–40% of GnRH neurons in rodents and sheep (Kriegsfeld et al., 2006; Smith et al., 2010; Poling et al., 2012; Rizwan et al., 2012; Ubuka et al., 2012) suggesting that RFRPs act centrally to control the reproductive axis. The RFRPs bind with high affinity to GPR147 (NPFF1R), a receptor coupled to adenylate cyclase via an inhibitory G protein (Mollereau et al., 2002). Autoradiographic analysis of GPR147 distribution in mice and rats indicated that the receptor is present throughout the hypothalamus (Gouarderes et al., 2004a,b). *In situ* hybridization showed that about 25–30% of GnRH neurons express *Gpr147* in various rodent species (Poling et al., 2012; Rizwan et al., 2012; Ubuka et al., 2012). Furthermore, RFRP-3 was found to alter the firing rate of GnRH-green fluorescent protein-tagged neurons, 41% of them being inhibited and 12% activated (Ducret et al., 2009). Altogether these observations support the hypothesis that RFRP-3 may exert its effects on reproduction directly via GnRH neurons. However, the peptide may also act indirectly, via upstream regulators of GnRH. Indeed, in the rat, RFRP-ir fibers are in contact with Kp neurons and a subpopulation of these Kp neurons express GPR147 (Rizwan et al., 2012).

In recent years, the effect of RFRPs, and particularly RFRP-3, was investigated on the reproductive function of various mammals. In male rats, central RFRP-3 suppresses all facets of sexual behavior and significantly reduces plasma levels of LH (Johnson et al., 2007; Pineda et al., 2010b). In female rats, chronic central infusion of RFRP-3 causes a dose-dependent inhibition of GnRH neuron activation at the LH surge peak and also suppresses neuronal activation in the AVPV region, which provides stimulatory input to GnRH neurons (Anderson et al., 2009). In ovariectomized mature rats, intravenous administration of RFRP-3 reduces plasma LH concentrations (Murakami et al., 2008). In ovine and bovine species, RFRP-3 administration inhibits gonadotrophin release, mainly acting at the level of the pituitary gonadotrophs (Clarke et al., 2008; Kadokawa et al., 2009; Sari et al., 2009).

Altogether these data indicate that RFRP-3, as opposed to Kp, is an inhibitor of the gonadotropic axis acting on GnRH neurons and/or pituitary gonadotrophs according to species. RFRP neurons are exclusively located in the mediobasal hypothalamus, a structure previously reported to be critical for the inhibition of reproductive function by melatonin in Syrian hamsters (Maywood and Hastings, 1995; Maywood et al., 1996). We thus investigated whether *rfrp* expression is regulated by the photoperiodic variation of melatonin and whether RFRP-3 regulates reproductive activity in the Syrian hamster.

## RFRP Expression is Down-Regulated by Melatonin in Short Photoperiod

In male Syrian hamsters, we observed that the levels of *rfrp* mRNA and RFRP-ir are markedly down-regulated in SD-adapted compared to LD-adapted animals, with no significant daily variation in both photoperiods (Revel et al., 2008). The higher level of *rfrp* expression in LD sexually active hamsters appeared to be in contradiction with the reported inhibitory effect of RFRP-3 on LH secretion, but our finding in the Syrian hamster was confirmed in another study (Mason et al., 2010). Furthermore, a similar down-regulation of *rfrp* expression and RFRP content was reported in the hypothalamus of other seasonal rodents, like Siberian (Revel et al., 2008; Ubuka et al., 2012) and European (Simonneaux and Ancel, 2012) hamsters and the Jerboa (Janati et al., 2013). In Syrian and Siberian hamsters, RFRP-ir fiber density and their appositions to GnRH neurons were also lower in SD as compared to LD conditions (Mason et al., 2010; Ubuka et al., 2012). Notably, RFRP-expressing neurons and RFRP-ir contact onto GnRH neurons are lower in SD conditions in the sheep as well (Dardente et al., 2008; Smith et al., 2008a). Altogether these observations indicate that the SD down-regulation in RFRP expression is conserved among seasonal species, irrespective of whether they are LD or SD breeders.

In male Syrian and Siberian hamsters, the SD down-regulation in *rfrp* expression is not a consequence of decreased levels of circulating sex steroids since neither testosterone implants in sexually inactive SD hamsters, nor castration in LD-adapted hamsters altered the levels of *rfrp* mRNA (Revel et al., 2008; Mason et al., 2010; Ubuka et al., 2012). In the ewe as well, the photoperiodic variation in RFRP expression appears independent of estrogen levels (Smith et al., 2008a). This lack of a major sex steroid feedback effect on *rfrp* expression is in agreement with most studies conducted in rats and mice (Quennell et al., 2010; Poling et al., 2012). Of note however, one study reported that E_2_ inhibits RFRP expression in mouse hypothalamus (Molnár et al., 2011). Notably, in both Syrian (Revel et al., 2008) and Siberian (Ubuka et al., 2012) hamsters, pinealectomy before transfer of hamsters to SD conditions, a protocol which prevents the SD-induced inhibition of reproductive activity, prevented the decrease in *rfrp* expression. Conversely, melatonin injections in LD-adapted hamsters, a protocol known to inhibit reproductive activity, induced a marked decreased in *rfrp* expression (Revel et al., 2008; Ubuka et al., 2012). Thus, in these species, melatonin is clearly driving the SD inhibition of RFRP expression, but whether this is due to a direct effect of the pineal hormone is yet unclear. Interestingly, melatonin acts directly on Mel1c receptors to increase GnIH expression in the quail paraventricular hypothalamus (Ubuka et al., 2005). In rodents, melatonin binding sites were found in the mediobasal hypothalamus of the Syrian hamster (Maywood and Hastings, 1995) but not in the Siberian hamster. Therefore, further studies are required in mammals to establish which of the hypothalamic and/or pars tuberalis melatonin receptors are involved in the photoperiodic regulation of RFRP expression.

In all seasonal species tested to date, the number of RFRP neurons and projections to GnRH neurons are decreased in SD conditions. Furthermore, manipulations of melatonin or sex steroid levels indicate that the SD down-regulation is driven by melatonin in a sex steroid-independent manner. In the SD breeding sheep, the increased expression of RFRP in LD is in line with its inhibitory effect. Several studies reported that RFRP-3 inhibits LH secretion in the ewe via both central (GnRH) and peripheral (pituitary gonadotropes) sites of action (Clarke et al., 2008; Smith et al., 2008a, 2012; Sari et al., 2009). In the LD breeding hamster however, the elevated RFRP expression in LD appeared contradictory with the hypothesized inhibitory action of the peptide. Actually, we (Ancel et al., 2012) and others (Ubuka et al., 2012) demonstrated in the male Syrian and Siberian hamster that RFRP-3 can activate the gonadotropic axis. In the male Syrian hamster, icv injection of RFRP-3 in LD-acclimated animals activates GnRH neurons and increases gonadotropin and testosterone secretion. This stimulatory effect of centrally applied RFRP-3 on LH secretion could not be duplicated by a peripheral administration *in vivo* or on cultured pituitaries, indicating that RFRP-3 is acting centrally, upstream of the gonadotropic axis. In the male Siberian hamster, central infusion of RFRP-3 increases LH secretion in SD conditions, whereas it is inhibitory in LD conditions. Interestingly, another study in ovariectomized female Syrian hamsters reported an inhibitory effect of central infusions of GnIH (Kriegsfeld et al., 2006). However, using the same model of ovariectomized female Syrian hamsters we found no effect of RFRP-3, possibly because in our study the circulating level of LH was kept at a low level (Ancel et al., 2012). It might be worth analyzing the effect of RFRP-3 in female rodents at different stages of their estrous cycle, where the circulating level of LH shows physiological variations. Altogether these findings indicate that RFRP-3 induces remarkable species- and gender-dependent effects on the reproductive axis.

In an attempt to evaluate whether the photoperiodic variation in RFRP expression is critical for the seasonal timing of reproductive activity, we tested the effect of chronic RFRP-3 administration in SD-adapted male Syrian hamsters. Actually, we observed that a 5-week icv infusion of a low dose of RFRP-3 was able to reactivate testicular function, to levels comparable to those observed in LD-adapted hamsters (Ancel et al., 2012). Importantly, we reported that this RFRP-3-induced reactivation of reproductive function was associated with a significant increase in *Kiss1* expression the ARC.

Taken together, these studies indicate that the SD conditions, via an increased secretion of melatonin, inhibit RFRP expression in the mediobasal hypothalamus of various seasonal breeders, irrespective of their seasonal physiology. Of interest however, the effects of RFRP-3 diverge among seasonal breeders, being stimulatory in LD breeders like hamsters and inhibitory in SD breeders like sheep. Therefore, it is tempting to speculate that RFRP neurons may play a key role in discriminating between LD and SD breeders. In order to fully understand the role of RFRP-3 in the seasonal control of reproduction, further experiments will have to be carried out in other species with particular attention to the animal gender.

## Do Kisspeptins and RFRPs Interact with Each Other to Synchronize Reproductive Activity with Seasons?

Both Kp and RFRP-3 regulate reproductive activity mainly via an action on GnRH neurons. Clearly, Kp and RFRP-3 act independently on the gonadotropic axis since RF9; a putative RFRP-3 antagonist is capable of increasing LH in mice devoid of Kiss1r (García-Galiano et al., 2012). However, only a few neuroanatomical and functional studies have been performed to examine whether both neuropeptides may interact with each other or via each other to control GnRH neuron activity.

In a variety of mammalian species both Kp and RFRP fibers project onto GnRH neurons and both Kiss1r and GPR147 receptors are expressed in GnRH neurons. According to studies and species, about 80–90% of GnRH neurons are targeted by Kp-ir fibers, express Kiss1r, and respond to a direct action of Kp (Irwig et al., 2004; Han et al., 2005) while 25–40% of these neurons are contacted by RFRP-ir fibers, express GPR147, or respond to a direct action of RFRP-3 (Kriegsfeld et al., 2006; Ducret et al., 2009; Poling et al., 2012; Rizwan et al., 2012; Ubuka et al., 2012). Therefore, there is a significant probability that both peptides may (inter)act on the same GnRH neurons. Highly selective pharmacological tools must still be developed to help disclose Kp and RFRP-3 interactions on GnRH neurons. This might be particularly critical to coordinate the LH surge where Kp and RFRP-3 have been reported to display opposite effects (Adachi et al., 2007; Gibson et al., 2008; Pineda et al., 2010b; Khan and Kauffman, 2012) on the gonadotropic axis of female rodents. This might also be of importance for the seasonal regulation of reproduction, particularly to discriminate between LD and SD breeders.

On the other hand, Kp-ir fibers (Clarkson et al., 2009; Desroziers et al., 2010) and a low density of Kiss1r (Lee et al., 1999; Herbison et al., 2010) are reported in the dorsomedial hypothalamus of rats and mice. Furthermore, our present data confirm the expression of Kiss1r in mediobasal hypothalamus of the Syrian hamster (Figure 1). Alternatively, RFRP-ir fibers and RFRP binding sites are reported in the ARC of various rodent species (Gouarderes et al., 2004b; Kriegsfeld et al., 2006). This indicates that the RFamide peptides may feedback on each other. Recently, an interesting study showed that about 20% of AVPV Kp neurons from proestrous female mice were contacted by RFRP-3 fibers, and that 9–16% of these neurons expressed GPR147 (Rizwan et al., 2012).

In our early experiment in the Syrian hamster, we did not check whether the stimulatory effect of Kp on the reproductive axis (Revel et al., 2006) had an effect on RFRP neurons. Conversely, in a more recent study, we observed that an acute central injection of RFRP-3 in the Syrian hamster induced c-Fos not only in GnRH neurons but also in ARC cells, although these were not Kp neurons. Nevertheless, the chronic administration of RFRP-3, which rescued reproductive activity in SD-adapted hamsters, induced a significant increase in ARC Kp expression (Ancel et al., 2012). This suggests that RFRP-3 may actually regulate ARC Kp expression, although in an indirect manner, to participate in the seasonal control of reproductive activity in the Syrian hamster. In order to test this hypothesis, it seems essential to carry out a detailed mapping of *Gpr147* in the Syrian hamster hypothalamus and design highly selective RFamide receptor ligands.

## Conclusion

**Figure 3:**
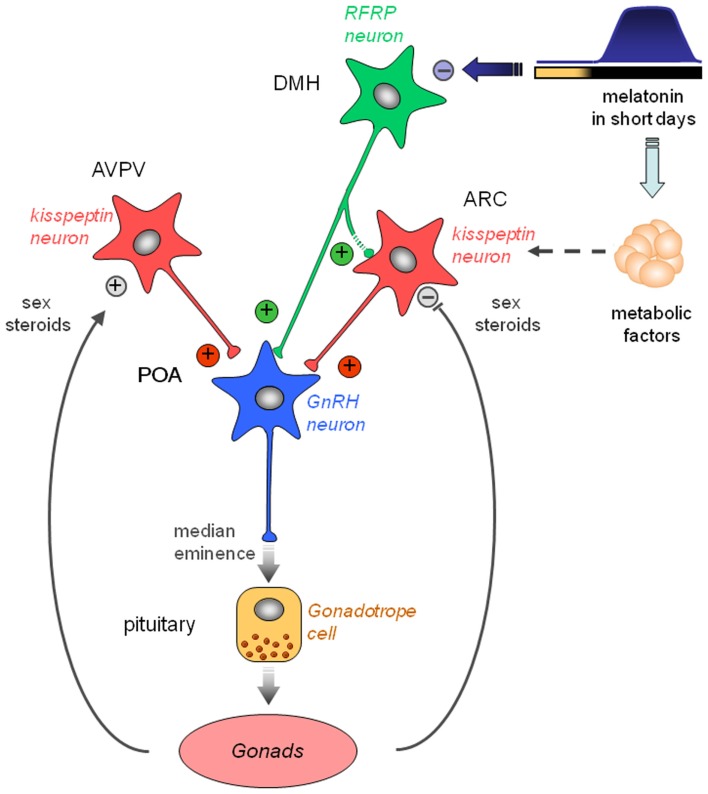
**Working model indicating how the photo-inhibitory melatonergic message in short day conditions is integrated in the hypothalamus to further regulate the gonadotropic axis of the male Syrian hamster**. GnRH neurons located in the preoptic area (POA) project at the median eminence to release GnRH which stimulates the release of pituitary gonadotropins downstream. GnRH neuron activity is regulated upstream by two populations of peptidergic neurons, the kisspeptin neurons located in the anteroventral periventricular (AVPV) and arcuate (ARC) nuclei, and the RFRP neurons located in the dorsomedial hypothalamus (DMH). Kisspeptin expression is up-regulated in the AVPV and down-regulated in the ARC by sex steroids. Of note, female AVPV contains a much higher number of kisspeptin neurons which are critical for the LH surge. ARC kisspeptin is regulated by metabolic factors and inhibited indirectly by melatonin. Kisspeptin is released at the level of GnRH cell bodies and nerve endings to induce GnRH secretion. RFRP neurons project to the GnRH neurons and possibly toward the ARC kisspeptin neurons. Although usually described as a gonadotropic inhibitor in other species, RFRP-3 induces a clear increase in GnRH neuron activity, kisspeptin expression, and testosterone production in the male Syrian hamster. In short days, the larger production of melatonin inhibits RFRP expression in a sex steroid-independent manner. This in turn reduces ARC kisspeptin expression and decreases gonadotropin secretion. Thus, RFRP neurons appear pivotal for the melatoninergic regulation of reproductive activity in seasonal rodents, although the pineal hormone may not act directly on RFRP neurons.

Several studies have now reported that Kp and RFRP-3 are regulated by photoperiod/melatonin and play a critical role in the regulation of seasonal reproduction. Kp are always very potent activators of the gonadotropic axis, ultimately able to reactivate reproduction of photoinhibed animals, either LD or SD breeders. Importantly, variation in Kp expression appears to depend on the species’ seasonal physiology, probably as a results of Kp sensitivity to a large array of endocrine and metabolic signals, particularly melatonin, leptin, and sex steroids, which all are critical for the regulation of seasonal breeding. In contrast, RFRP-3 expression appears to be reproducibly down-regulated in SD in the various species studied up to date. In Syrian and Siberian hamsters, the SD inhibition is driven by melatonin and independent of sex steroid feedback. Although RFRP-3 is considered an inhibitor of gonadotropin secretion in rats, mice, and sheep, it is able to stimulate the gonadotropic axis in male Syrian and Siberian hamsters. Furthermore, a chronic infusion of the peptide in SD-adapted hamsters restores an LD profile of both ARC Kp expression and reproductive activity. From these findings, we propose a working model for the seasonal regulation of reproduction in the male Syrian hamster (Figure 3). In this model, RFRP neurons of the dorsomedial hypothalamus might play a pivotal role in the melatoninergic control of seasonal reproduction. RFRP neurons might be the primary central target for the inhibitory action of the SD melatonin signal. However, melatonin could act in an indirect manner, via the recently well-described pars tuberalis TSH/basal hypothalamus T3 production. RFRP-3 might regulate GnRH neuronal activity directly and/or indirectly via Kp neurons. In addition, the melatonin-driven TSH/T3 signal or melatonin itself might regulate Kp expression indirectly via other seasonally regulated metabolic or gonadic endocrine signals.

## Conflict of Interest Statement

The authors declare that the research was conducted in the absence of any commercial or financial relationships that could be construed as a potential conflict of interest.

## Abbreviations

ARC, arcuate nucleus; AVPV, anteroventral periventricular nucleus; FSH, Folliculo-stimulating hormone; GnIH, gonadotrophin-inhibitory hormone; GnRH, gonadotrophin-releasing hormone; GPR147, RFRP receptor; Kiss1R, kisspeptin receptor, also known as GPR54; Kp, kisspeptins; LD, long days; LH, luteinizing hormone; POA, preoptic area; RFRP-3, Arg-Phe amide related peptide-3; SD, short days.
